# Prevalence, Risk Factors, Pathophysiology, Potential Biomarkers and Management of Feline Idiopathic Cystitis: An Update Review

**DOI:** 10.3389/fvets.2022.900847

**Published:** 2022-06-21

**Authors:** Chengxi He, Kai Fan, Zhihui Hao, Na Tang, Gebin Li, Shuaiyu Wang

**Affiliations:** Small Animal Department, College of Veterinary Medicine, China Agricultural University, Beijing, China

**Keywords:** feline idiopathic cystitis, stress, etiopathogenesis, biomarkers, multimodal environmental modification

## Abstract

Feline idiopathic cystitis is a widespread disease in small animal clinics, which mainly presents with urinary signs like dysuria, stranguria, hematuria, pollakiuria, and periuria. The etiopathogenesis of the disease may involve interactions between the environmental stressors, neuroendocrine system and bladder of affected cats. Diagnostic biomarkers have not been tested in clinical studies though they are theoretically feasible, and since the clinical signs of the disease assemble those of other feline lower urinary diseases, its diagnosis is a procedure of exclusion. The primary treatment of the disease is long-term multimodal environmental modification (or enrichment) while anti-anxiety drugs and nutritional supplements are recommended for chronic recurrent cases. Still, many medicines need to be evaluated for their efficacy and safety. This review aims to provide readers with a comprehensive understanding of feline idiopathic cystitis by summarizing and updating studies concerning the prevalence, risk factors, etiological hypotheses, diagnostic procedures, possible treatments, and prognosis of the disease.

## Introduction

Lower urinary tract signs (LUTS), including dysuria, stranguria, hematuria, pollakiuria, and periuria (inappropriate urination), are commonplace in cats, and inappropriate elimination is responsible for millions of cats being relinquished to shelters or euthanized (1). In the 1970s, the term feline urologic syndrome (FUS) was popularly used to represent all medical problems of cats having LUTS (2–4), regardless of different sites of involvement, different combinations of clinical signs, and fundamentally different causes (5). It had been used in later decades and nowadays, the term feline lower urinary tract disease (FLUTD) is generally the synonym of FUS, which also encompasses various disorders with heterogeneous causes that affect the urinary bladder and/or urethra of cats (6), so the cats with FLUTD show one, several or all LUTS. As early as 1984, Osborne et al. had recommended that ambiguous terms like FUS should be replaced with specific diagnostic terms relating to sites, causes, morphologic changes, and pathophysiologic mechanisms (7). At present, according to diverse causes, FLUTD can be classified into feline idiopatic cystitis (FIC), urolithiasis, bacterial urinary tract infection (UTI), neoplasia, anatomic malformations, and iatrogenic, behavioral, metabolic, or neurologic problems (8, 9). If exact causation is not found after thorough evaluation, a diagnosis of FIC can be made (10, 11). FIC also refers to feline interstitial cystitis in some studies, and the terms idiopathic cystitis and interstitial cystitis are often used interchangeably (9). The word “interstitial” was chosen due to the similarities FIC has with interstitial cystitis /bladder pain syndrome (IC/BPS) in humans (12), such as clinical signs, recurrence tendency, comorbidities, and correlations with stress (10, 13). Because of the complexities of the disease and its relation with psycho-neuro-endocrine factors, IC/BPS is also termed as medically unexplained syndrome (14), central sensitivity syndrome (15), or functional somatic syndrome (16). Likewise, although some theories suggest that one or several interrelated mechanisms may induce lower urinary tract (LUT) dysfunction as well as comorbidities in other body systems and organs, the precise etiopathogenesis of FIC has not yet been defined, and thus Buffington has proposed to use “pandora syndrome” as an interim name to substituted FIC until the most medically appropriate nosological term is identified (13). Tentative criteria for diagnosis of a “pandora syndrome” include exhibiting clinical signs both in LUT and other organ systems; waxing and waning of clinical signs associated with stressful events; and resolution of severity of clinical signs following effective environmental enrichment (17).

However, since FIC was the term used at the time many papers were published, we still use FIC instead of “pandora syndrome” in this article to avoid confusion.

## Epidemiology

### Prevalence of FIC

**Figure 1:**
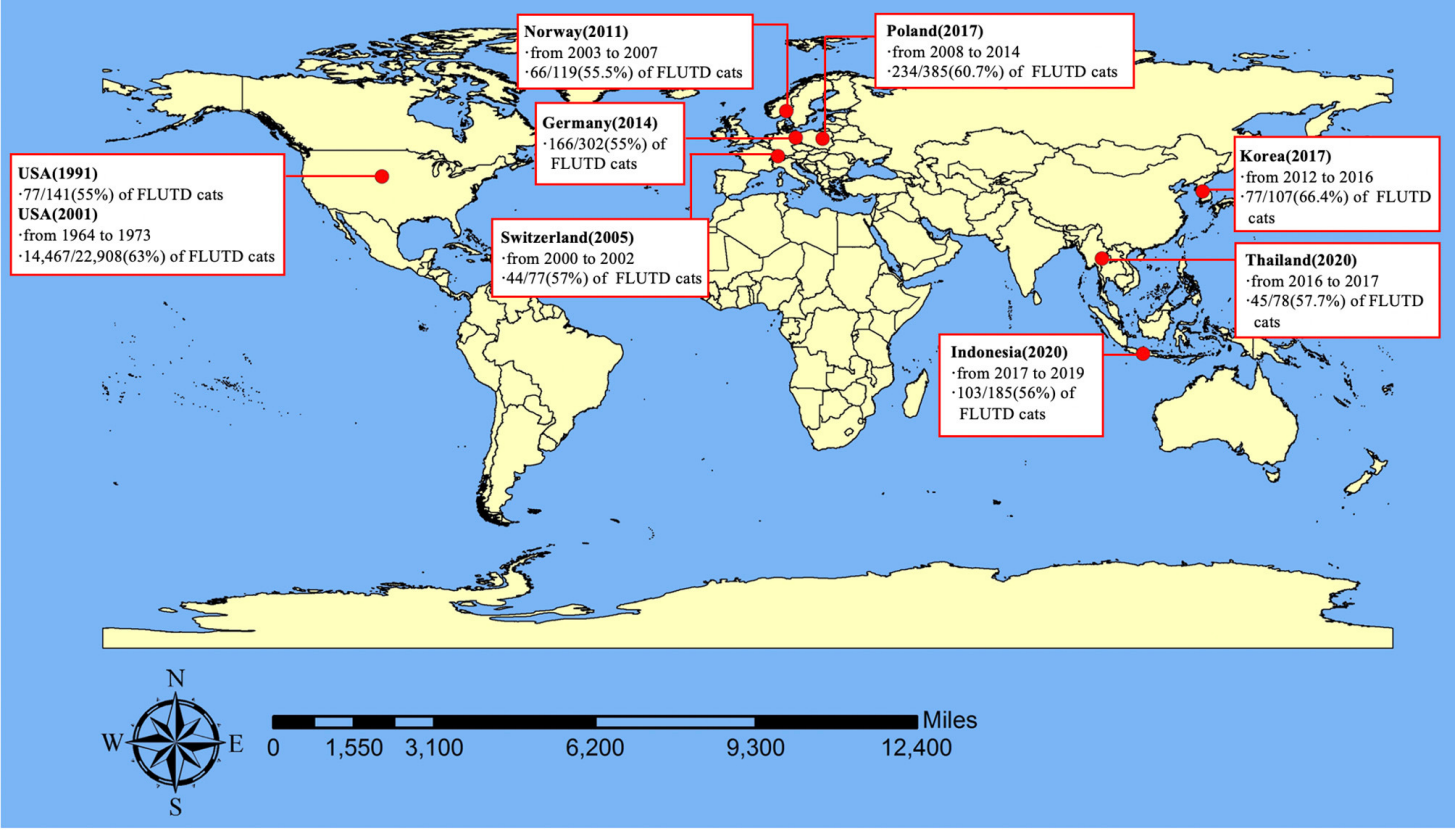
Prevalence of FIC.

FLUTD is a spontaneous syndrome commonly seen in small animal practice, and its prevalence was reported to be 1.5% in the United States (18), 4.4% in British (19), 2.67% in Korea (20), and 2.2% in Thailand (21). These findings, combined with some results mentioned in Sparkes's review and some unpublished data (22), confirm that FLUTD occurs commonly, accounting for ~1.5–4.5% of the whole feline caseload in veterinary clinics. The prevalence of cats classified as FIC in FLUTD cats was reported to be 55% in 1991 (23), and 63% in 2001 in the United States (5), and has been estimated to be 57% in Switzerland (8), 55% in Germany (24), 55.5% in Norway (11), 60.7% in Poland (25), 66.4% in Korea (20), 57.7% in Thailand (21), and 56% in Indonesia (26). Therefore, it can be concluded that the prevalence of FIC among cats with FLUTD ranged from 55% to around 67% and FIC is the most frequent cause of FLUTD (Figure 1). FIC can be obstructive or non-obstructive, and non-obstructive FIC is the dominant type, which can display in three ways: a single acute seemingly self-limiting episode (80–90%), frequently recurring episodes (2–15%), and persistent forms (2–15%) (27).

### Risk Factors of FIC

The risk factors of FIC differ across countries due to environmental factors, husbandry practice, cats' lifestyle and other cat-related factors (28, 29). Also, many studies have discovered that stress may play a role in the development of FIC (30–33). Short-term or long-term exposure to unusual external events and unpredictable factors that act as stressors can make cats feel nervous and fearful (28, 31, 33), leading to FIC and demonstration of LUTS (34).

Almost all studies agree that male cats have an increased risk to develop FIC (11, 21, 25, 31). Some studies have proposed that castration (neutering) is associated with increased risk for FIC (5, 20, 21). Urethral obstruction (UO) refers to the situation when cats could not urinate as a result of functional obstruction (idiopathic obstruction) or a physical obstruction, such as mucous plugs or calculi within the urethra (35). UO occurs with higher frequency in castrated male cats compared with female cats because of their extremely narrow penile urethra (5, 9). Some studies indicated that cats with FIC are more likely to be purebred (3, 31), longhaired (36), and middle-aged (25, 37). One study revealed that cats between 2 and 7 years old have an increased risk of developing FIC compared with cats below 1 year old (5). Also, being overweight has been proved to be significantly related to the incidence of FIC in many studies (20, 31–33). In addition, many studies have found that cats with FIC are more likely to eat dry food (21, 36), and drink less water than the control cats (32). Furthermore, there appears to be a correlation of number of litter box availability to number of cats in household (21, 32, 36). Additionally, lots of studies have suggested indoor confinement of cats is prominently associated with the occurrence of FIC (11, 20, 32, 36, 38). Cats with FIC have less activities and less hunting behaviors than the control group (32, 36) and are more likely to lack access to elevated vantage points (20, 33). What's more, some studies have found that cats with FIC are more easily to feel fearful, anxious and stressful than the control group (11, 33), for example, they are disposed to escape and hide from unfamiliar visitors (32). Also, cats who live with other cats or animals are prone to develop FIC, especially when conflicts persist between the vulnerable cat and other family members (20, 31, 32). Under stressful circumstances, the sound, smell, appearance and actions of another cat will be sources of threat and anxiety, which could easily trigger FIC (30, 31). Additionally, events which cats perceive as less safe and comfortable, such as the position of food dishes, water bowls and litter boxes (33), and changes in cats' life like house moving (32, 36), may be significantly associated with the occurrence of FIC. Other risk factors, such as seasonal variations (36), a meal fed routine (33), using clumping litters, and sharing food and water bowls with other cats in the household (20), may also connect to the rising odds of FIC in some ways.

Knowing risk factors of FIC can help veterinarians and pet owners to take care of potential stressors and tailor an environmental enrichment schedule (discussed later) for cats with FIC, or even better, optimize the environment early in cats' life, and thus prevent LUTS associated with FIC (39).

## Pathogenesis

Based on available evidence, FIC represents a complex syndrome with multiple causes (10), and seemingly separate but potentially interrelated mechanisms between the environment, neuroendocrine system and bladder of affected cats exist (17). According to Buffington (13), possible causes of FIC could be divided into three major groups with regards to the origin of etiological factors: abnormalities existing within the LUT lumen are termed local external abnormalities; changes occurring in the LUT itself are classified as intrinsic abnormalities; and abnormalities originating from other organ systems that may contribute to LUT dysfunction are termed internal abnormalities.

### Local External Abnormalities

Bacterial infection was once thought to be a cause of FIC because the rapid onset and clinical signs of FIC are similar to UTI (40), and antimicrobic medicines were thought to resolve clinical signs (10). But in fact, some forms of FIC are self-limiting, which means that the affected cats will undergo spontaneous remission in 5–10 days irrespective of whether or not they have received therapy (10, 37). Similarly, the hypothesis that urinary crystals and matrix plugs were the cause of FIC was previously proposed, but then proved to be unconvincing (10). However, although not the common cause of FIC, microbes and struvite crystalluria may have associations with LUTS of the syndrome in some aspects (41, 42).

Viruses, such as feline calicivirus (FCV) (43), feline foamy virus (FFV), and bovine herpesvirus type 4 (BHV-4), have been suggested to be causative agents for FIC, at least in some cases (10, 44). The high prevalence of FFV and BHV-4 antibodies presenting in FIC-affected cats, and the exacerbated severity of clinical signs in cats with FIC infected with BHV-4 and FCV enhance the hypothesis (10). Moreover, FCV-U1 and FCV-U2 are two strains isolated only from urine of cats with FIC (45), and this suggests their unique role in the pathogenesis of the disease (44). Nevertheless, although some functional components, such as junctional adhesion molecule 1 and α 2,6-linked sialic acid of FCV have been examined (46, 47), additional studies defining the pathogenic role of viruses in the formation of FIC are desperately wanted (10).

### Intrinsic Abnormalities

The bladder wall is composed of urothelium, submocosa, and muscularis. The urothelium is stratified and it contains three layers: basal, intermediate, and superficial apical layer, and the latter one contains mainly large hexagonal cells (also known as umbrella cells) (48). Superficial urothelial cells are responsible for maintaining the bladder barrier, which largely depends on three elements: high resistance tight junctions between apical membranes of adjacent urothelial cells; a layer of glycosaminoglycans (GAGs) located on the luminal surface of urothelial cells; and the unique lipid and protein composition of urothelial cell membranes (49). Anything that alters structural or functional characteristics of GAGs or tight junctions, disrupts active transport mechanisms, and/or directly injures urothelium may result in malfunction of the bladder barrier (10). In cats with FIC, decreased urinary concentration of total GAGs (50, 51) and some components of GAG layers, such as chondroitin sulfate (51), perlecan, biglycan, and decorin (49), have been documented. Also, the expression of zonula occludens-1 (ZO-1), a constituent of the tight junction complex (52), has been reported to reduce in bladder biopsies of cats with FIC. Moreover, abnormal expressions of cell differentiation markers, like uroplakin, keratin 20, E-cadherin (49), and AE-31 (52), have been identified in cats with FIC, which may indicate their adaptation to the impaired, permeable bladder. What's more, denudation of umbrella cells, thinning of the transitional cell epithelium (52), submucosal edema, hemorrhage, neovascularization, mastocytosis, mononuclear inflammatory cell infiltration, and fibrosis have also been recorded in bladder biopsies of cats with FIC (10, 52–54).

### Internal Abnormalities

It has been found that cats with FIC appear to be more sensitive than healthy cats to environmental, psychological, and pathological stressors, and more likely to conduct chronic stress responses (30, 36), which may link to, just like in human beings, mood alteration, pain symptom (55, 56), immune activation, proinflammatory cytokine release (57), and finally and most importantly, dysfunction of homeostatic regulatory systems (34, 55, 58). So we can conclude that systemic psycho-neuro-endocrine disorders may be involved in the pathogenesis of the disease (10, 30, 59–61). The reason why LUTS predominate in cats with FIC is probably that Barrington's nucleus, the pontine micturition center (62), is anatomically located near the fear pathway, and thus places the bladder at increased risk for stimulation during stress responses (34).

#### The Relationship Between Stress Stimuli and Bladder Abnormalities

The possible mechanisms of stress-induced LUTS in cats with FIC is by triggering central dysregulation of autonomic neurons that either regulate bladder contraction or directly compromise urothelium (63, 64).

In cats with FIC, literature has found that increased sympathetic activity can cause the stimulation of C-fibers, the unmyelinated pain sensors located in bladder submucosa (9). Also, the release of substance P (SP), a neurotransmitter (9, 13), and the expression of SP receptors in the bladder of cats with FIC is significantly increased (13). Additionally, various chemical signaling molecules, such as prostaglandins, nitric oxide (NO) (65), acetylcholine (Ach) (66, 67), and adenosine triphosphate (ATP) (22, 68–70), could also activate afferent neurons and adversely affect bladder function of cats with FIC (10, 71). Studies have identified the increased release of NO is mainly from the urothelium and afferent nerves in bladder strips (65), and have proposed the possibility that the change may result from the raised expression of norepinephrine (NE) from adrenergic nerves in the bladder or increased concentration of catecholamines from the adrenal medulla by acting on β-adrenergic receptors in the urothelium (13, 64). In addition, increased levels of endogenous or circulating serotonin have been reported in cats with FIC (72), which can elevate the concentration of non-neuronal Ach (67), and then increase the distention-evoked ATP release from the urothelium by stimulating urothelial muscarinic receptors (68, 72). ATP has been thought to play a role in bladder afferent sensitization, possibly by activating P2X3/2 purinergic receptors located on sensory terminals in the mucosal layer (73). What's more, neurogenic inflammation in the bladder of cats with FIC may be triggered by histamine and other bioactive mediators released by independently activated mast cells in adjacent to neuropeptide-containing sensory neurons (74, 75). Increased activity of the sympathetic nervous system (SNS) and various chemicals described above could all contribute to the compromised urothelial barrier in cats with FIC, which can offer potassium and other toxic irritants in the urine greater access to sensory afferent neurons innervating the urothelium (76), and in turn worsen the pain and LUTS in cats with FIC (52, 77).

#### The Role of Neuroendocrinal Abnormalities in FIC

In addition to LUTS, cats with FIC may also present variable combinations of comorbid disorders (28, 30, 63). There is a theory that clinical signs of cats with FIC may result from a common underlying cause (13). Internal abnormalities which could induce a cascade of neural, endocrine and immune events that likely affect multiple tissues in different body systems show the most promise (78). The anatomy and physiology of sensory neurons in both central and peripheral nervous systems are altered, and specific evidence is shown below.

Capsaicin-responsive afferent neurons in bladder (13) and in dorsal root ganglion throughout the lumbosacral (L4-S3) spinal cord (79) of cats with FIC have been reported to be 30% larger than normal cats (69), and also, these neurons have been found to exhibit desensitizing K^+^ currents, increased electrical excitability in response to depolarizing current pulses, and express changed receptor sites and more neurotransmitters and inflammatory mediators, which may contribute to bladder pain in cats with FIC (13, 79). These findings suggest aberrances of sensory neuron function are widespread (80), and may further explain the phenomenon that manifestations of FIC sometimes extend beyond the bladder (63).

Cats with FIC have increased responsiveness to unexpected auditory stimuli, termed acoustic startle reflex (13, 30), especially during stressful conditions (81). This also suggests some forms of internal abnormalities must exist in the diseased cats.

Activation of the SNS, which is responsible for arousal, analgesia, vigilance, and visceral responses to stress, plays an important role in the pathophysiology of FIC (13, 30). The pontine locus coeruleus (LC) contains the largest number of noradrenergic neurons, and is the most important source of NE in the central nervous system (CNS) (13, 29, 82). Afferent input, such as perception of threat and bladder distention, activates neuronal activity in the LC, which then leads to the excitatory stimulation in the bladder (9, 29, 83). The increased tyrosine hydroxylase (TH) immunoreactivity in the LC and the paraventricular nucleus of cats with FIC has been identified (60), suggesting increased catecholamine synthesis. Affected cats also have increased concentrations of plasma catecholamines and their metabolites (59), including epinephrine, NE, dihydroxyphenylalanine, and dihyroxyphenylglycol (64, 84), during stressful situations, and the condition persists even when these cats have acclimated to stress (9, 80). Corresponding increase of TH and NE in cats with FIC indicates an exaggerated sympathetic outflow, mainly result from altered activity in LC, Barrington's nucleus and paraventricular nucleus caused by physical and mental stressors (29, 30).

Decreased functional sensitivity of α2 adrenergic receptors (α2-ARs) has been identified in cats with FIC (85). Desensitization in α2-ARs responses may be a result of chronic exposure to the elevated plasma concentrations of their agonist, catecholamine (85, 86). Normally, α2-ARs are presented to restrain NE release in the LC of the brain stem and inhibit transmission of nociceptive input to the brain in the spinal cord (85, 87, 88). However, as the activity of α2-ARs is downregulated by NE in FIC-affected cats, signs of pain may occur.

Adrenal glands of cats with FIC have been found to possess significantly reduced volume and weight and show weakened responses to adrenocorticotropic hormone (ACTH) (61). These results, when combined with observations of increased concentrations of corticotropin releasing factor (CRF) in stressed cats with FIC, which then trigger an increased ACTH response and a decreased cortisol response (64), suggest the presence of mild primary adrenocortical insufficiency in cats with FIC (13, 61). Under normal circumstances, cortisol should modulate sympathetic neural outflow, as well as give a negative feedback on the hypothalamus and adrenal glands to inhibit its own release (13, 89). However, in susceptible cats suffering from chronic stress, the uncoupling of SNS activity from the hypothalamic-pituitary-adrenal (HPA) axis characterized by exaggerated catecholamine release and blunted cortisol response has been documented (9, 17, 59, 61, 63, 64, 90, 91), which then results in increased sensory stimulation of the bladder and altered urothelial permeability (92).

**Figure 2:**
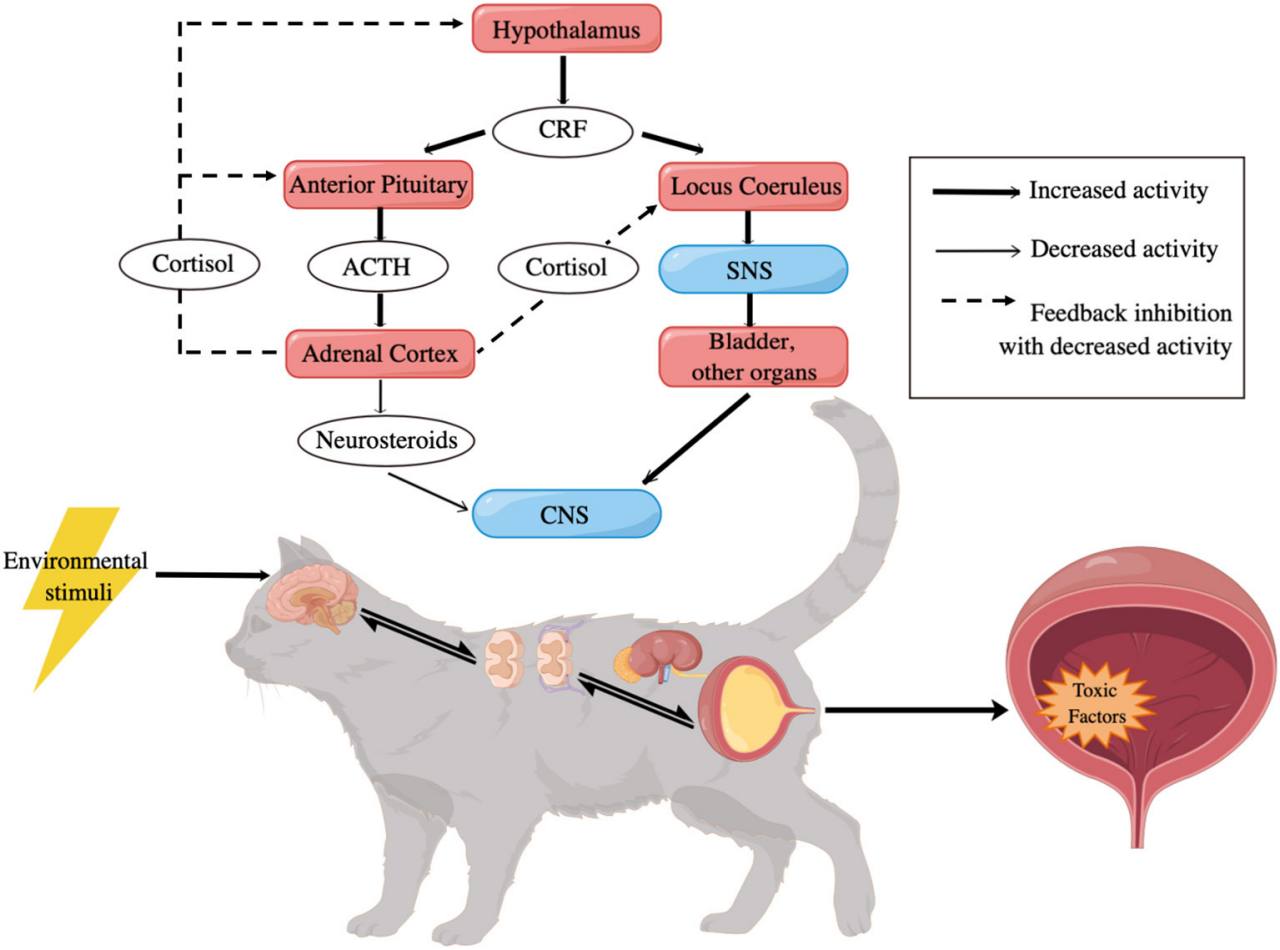
Potential pathogenesis of FIC [adapted from Hosutler et al. (9)]. CRF, corticotropin releasing factor; ACTH, adrenocorticotropic hormone; SNS, sympathetic nervous system; CNS, central nervous system.

Genetic predisposing factors (9), like epigenetic modulation of gene expression (EMGEX), may be involved in the process as well (90, 93). Early adverse experiences can alter the central threat response system (CTRS) (39) of cats in both their prenatal and postnatal life (63, 80, 94). When exposed to stressors that are harsh enough to induce intense stress responses, hormones can transmit across the placenta to influence the CTRS of the fetus in pregnant cats, or directly affect the CTRS of young individuals after their birth, resulting in enhanced SNS activity, along with enhanced ACTH production and low cortisol response (63, 95). It has been postulated that these cats are more vulnerable to stress factors, including intrinsic unpleasantness, unfamiliarity, the discrepancy from expectation and decreased capacity for control (34), and thus become more likely to develop FIC (Figure 2) (9).

## Diagnosis

Untill now, the pathogenesis of FIC has not been clearly defined, and no sensitive and specific test of FIC is clinically available (39, 96), so the diagnosis of FIC is still a procedure of exclusion, by ruling out other LUT-related disorders, such as urolithiasis, UTI, anatomic abnormalities, behavioral disturbances, and neoplasia (6, 64).

### Behavioral History

The diagnosis of FIC primarily relies on the clinical signs and behavioral history of the affected cats. To establish a medical diagnosis, marking and toileting problems should be excluded (97, 98). Cats with FIC may present with varying combinations of LUTS, including dysuria, stranguria, periuria, pollakiuria and macroscopic hematuria (13, 32). Also, cats with FIC may present relentlessness before, vocalization during, and excessive grooming after urinations (99), and these behaviors indicate stress and pain. The disease is a chronic problem, which may resolve itself temporarily but relapse frequently (6, 64), and the occurrence of the first and later episodes are commonly associated with events which the affected cats perceive as stressors. So, a detailed urinary history of the cat should be recorded, as well as other abnormal behaviors beyond the LUT and a thorough environmental history (17). Special attention should be given to potential stressors and risk factors as described above. That information could be gathered from pet owners through well-designed questionnaires (39).

### Physical Examination

For cats presenting with LUTS, abdominal palpation is required to test the cat's pain reaction and to roughly figure out the size, texture and inclusions of its bladder based on veterinarians' experiences. Complete and comprehensive physical exams should be conducted to detect comorbid abnormalities (13, 29). Some etiological hypotheses suggest, FIC may be caused by complex but interrelated neurological, hormonal, immunological and environmental mechanisms (78), and FIC may be a urinary manifestation of a systemic disorder (63).

### Imageological Evaluation

Diagnostic imaging is used to exclude other causes of FLUTD. Abdominal plain radiography can help identify radiopaque calculi (e.g., struvite, oxalate) of more than 3 mm in diameter (9). Additionally, contrast radiography, including retrograde contrast urethrography, positive and double-contrast cystography and urethrocystography, can help to detect small lesions, radiolucent calculi, neoplasia (9), and anatomic defects, such as urethral strictures, urachal diverticula, and urethrorectal fistula (17). Abdominal ultrasonography can also assist in identifying urinary calculi and crystals, bladder masses, and bladder wall thickness (100). Furthermore, uroendoscopy, including cystoscopy and urethroscopy, can provide visualized views of the urethral and bladder mucosa, and evaluate urinary lesions, stones, masses, and strictures more directly (9).

### Laboratory Evaluation

Laboratory examinations are of great importance to rule out other possibilities of FLUTD. The results of routine hematology and serum biochemistry of cats with FIC are often normal or show minor deviance, which is not specific for the diagnosis of FIC. Urine sediment analysis, along with urine culture and sensitivity test is highly recommended. Although significant differences in the urine pH, urine specific gravity, and amounts of epithelial cells in the urine sediment may not be observed between FIC and other categories of FLUTD (11), the urinalysis may identify hematuria and proteinuria in cats with FIC, and when pyuria is ascertained, a diagnosis of UTI can be made. However, when examining urine sediment under a microscope, care must be taken not to overinterpret the presence of bacteria. Some particulates like small crystals, cellular debris, and lipid droplets may exhibit Brownian motion and thus be misinterpreted as bacteria (9). Also, urine samples should be collected by sterile method and should not be refrigerated or stored for too many hours before examination to avoid false-positive or exaggerated results from appearing, for instance, crystals (9, 101, 102). Moreover, bladder biopsy and histopathology is the gold standards to diagnose neoplasia, while it is unnecessary for the clinical diagnosis of FIC because histopathologic abnormalities are not pathognomonic for FIC (9, 103).

### Potential Biomarkers

Many studies have examined potential biomarkers of FIC in urine, plasma, or serum specimen for a long time, and some competent candidates have emerged. In FIC-affected cats, chronic urinary or comorbid non-urinary inflammation, and perhaps activation of SNS, may induce systemic immune responses. In serum profiles of cats with FIC, pro-inflammatory cytokines interleukin 12 (IL-12), interleukin 18 (IL-18), fms-related tyrosine kinase 3 ligand (Flt3L) and chemokine C-X-C motif chemokine ligand 12 (CXCL12) were discovered to increase significantly (96). In urine samples of cats with FIC, an upregulation of fibronectin (53, 104) and a downregulation of an important growth factor, the trefoil factor 2 (TFF2) have been discovered (54). Fibronectin is a high-molecular-weight glycoprotein which has an important role in cell growth, differentiation, adhesion and migration (105). Fibronectin exists in locations of tissue inflammation and fibrosis (106). Fibrosis has been found in the bladder muscle layer and vascular walls of cats with FIC under histologic examination, and a high level of insoluble fibronectin in urine might result from its detachment from the leaky bladder barrier (53, 104). TFF2 deficiency may lead to impaired repairing abilities and susceptible immune response of the urothelium (54). Moreover, an increase of complement 4a (C4a), galectin-7, thioredoxin, nuclear factor-κB p65 (NF-κB p65), and p38 mitogen-activated protein kinase (p38 MAPK) has been identified, while I-FABP (also known as fatty acid-binding protein 2) has been found to decrease in the urine of affected cats as compared to healthy controls (107). Bladder inflammation in cats with FIC could activate the complement system and an increasing amount of C4a is allowed to leak into the urine through the damaged, hyper-permeable bladder wall (107). Galectin-7 plays a crucial role in wound healing and epithelialization (108–110), which explains its augmented expression in impaired bladder tissue and urine of affected cats (107). Thioredoxin is a redox-regulating protein (111), and in FIC cases, increased abundance of thioredoxin will be secreted by injured tissue cells into the urine, where it performs as a regulator of apoptosis (112) and a protector against oxidative stress (107). NF-κB p65 and p38 MAPK are two transduction molecules of thioredoxin, which follow the same increasing pattern of thioredoxin in urine of cats with FIC. Nevertheless, as the only protein that decreases in both tissue and urine samples of FIC-affected cats, the downregulation of I-FABP might be triggered by the decreased concentration of fibronectin in diseased, permeable bladder layers (53).

Histologically, FIC in cats resembles the non-ulcerative form of IC/BPS in human beings (52, 103), so some potential biomarkers of IC/BPS may play a similar role in FIC. In urine of IC/BPS patients, anti-proliferative factor (APF), hemoglobin-binding endothelial growth factor (HB-EGF), endothelial growth factor (EGF), nerve growth factor (NGF), and brain-derived nerve growth factor (BDNF) have all been proposed as biomarkers (113, 114). APF is a frizzled-related sialoglycopeptide which serves as a bladder epithelial growth inhibitor (10, 115–118), and its amount is significantly increased in the urine of IC/BPS patients (116, 117, 119). In addition, HB-EGF and EGF can stimulate urinary epithelial cells replication and proliferation, and their activities may be regulated by APF (119). In the urine of patients with IC/BPS, the quantity of HB-EGF is reduced whereas the value of EGF is raised (113, 119). In addition, an upregulation in synthesis and secretion of NGF and BDNF have been detected in urine specimens of IC/BPS patients in many studies (120, 121). NGF and BDNF are neurotrophins produced by the urothelium and smooth muscle cells in the bladder, and could sensitize afferents, leading to urgency, frequency and bladder pain in patients (114, 122–124). Moreover, significantly elevated levels of tyramine, the pain-related neurotransmodulator, and 2-oxoglutarate (also called α-ketoglutarate), which may retard growth of bladder epithelial cells, have been identified in urine of IC/BPS subjects (125). Furthermore, the serum concentration or plasma concentration of pro-inflammatory cytokine IL-1β, IL-6, and TNF-α and chemokine IL-8 has been detected to be significantly higher in people with IC/PBS than in controls (126, 127). What's more, compared to normal subjects, Tamm-Horsfall protein (THP) in IC/BPS patients has less sialylation and less overall glycosylation, leading to reduced protective effect against cationic toxic factors in urothelial cells (128, 129). Also, in biopsy specimens of patients with IC/BPS, increased E-cadherin staining and decreased ZO-1 staining have been detected (130, 131), which may suppress epithelial proliferation and differentiation in the bladder (118). Aberrant expression of several other urothelial growth-related factors, which contribute to the bladder integrity and impermeability (131), such as GAGs, glycoproteins (e.g., GP51, GP1) (132, 133) proteoglycan core proteins (biglycan, decorin, perlecan, and syndecan-1), keratins (134, 135), and uroplakins (e.g., III-delta4) (131, 136), have been revealed in IC/BPS cases as well.

All these findings suggest promising candidates for non-invasive biomarkers of FIC to define the presence and monitor therapeutic outcomes of the disease (96). However, many questions about their specificity, sensitivity, and as to whether or not they are cost and time effective, remain (137). Therefore, more studies are needed to find and bring ideal diagnostic markers into clinical utilization.

## Treatment

The specific etiopathogenesis of FIC has not been elucidated, and one cure-for-all treatment has not been established (17, 138). Therefore, the principle of treatment is to reduce duration, severity, and the recurrence of LUTS, as well as the risk for UO (17). Due to the chronic recurrent disease in a substantial number of patients (139), close cooperation between dedicated pet owners and veterinary staff is needed to successfully manage the affected cats (9, 29). Since the beginning of studying FLUTD, many agents or procedures have been recommended for management and prevention of FIC, however, few of them have been investigated in randomized, placebo-controlled, double-blinded experiments (37, 140, 141). This defectiveness, together with the self-limiting property of FIC, make the effectiveness of these treatments difficult to be assured (37, 140, 141). So, in this part, we will review studies about therapies of FIC, make a thorough examination of their efficacy and draw a conclusion.

### Pharmacological Therapy

#### Inefficient Drugs for FIC

FIC is unlikely to be caused by bacterial infection (142), so there is no reason and no use to administer antibiotics in FIC-affected cats (29, 37, 143, 144). Previous findings of the seeming usefulness of antibiotics in resolving the clinical signs of cats with FIC may actually be due to their spontaneous remission. Nevertheless, when urethral catheterizations or a perineal urethrostomy are conducted in cats with FIC with UO, antimicrobial agents are recommended as a preventative procedure to reduce the risk of bacterial UTI (29, 35).

In a randomized, double-blind, well-controlled, prospective clinical trial, the effect of prednisolone (1 mg/kg BID PO for 10 days) was assessed in 12 cats with FIC, but no difference of severity and duration of the disease has been discerned between cats treated with prednisolone and cats treated with placebo (145). Thus, prednisolone is not recommended as an effective treatment for cats with FIC.

#### Drugs for Acute FIC

Acute FIC refers to the first and sudden onset of the disease, or an acute episode after a relatively long interval.

Smooth and skeletal muscle fibers are responsible for generating urethral tone and causing urethral spasms in cats with FIC (37), and antispasmodic drugs might be beneficial to reduce the severity of LUTS and the risk of UO (37). Smooth muscle antispasmodics include acepromazine, prazosin, and phenoxybenzamine while skeletal muscle antispasmodics contain dantrolene (37, 99). All these medicines should be tapered over multiple days (37). Propantheline, an anticholinergic drug, can also relax bladder spasms, and thus help to treat the urinary incontinence associated with FIC (144). However, flavoxate and oxybutynin are currently used more frequently than propantheline. These recommendations are based on theory and previous experiences, and should be seriously tested in the future.

Opioid analgesics, including butorphanol, buprenorphine, and fentanyl (29), and non-steroidal anti-inflammatory drugs (NSAIDs), such as carprofen, ketoprofen, meloxicam, piroxicam, and robenacoxib (9, 17), seem to be beneficial in relieving bladder pain and reducing the severity of clinical signs in cats with FIC (9, 37). Theoretically, opioids perform analgesic effect by combining with opioid receptors and inhibiting the release of SP, and NSAIDs produce anti-inflammatory and analgesic effect by inhibiting the activity of cyclooxygenases and decreasing the synthesis of prostaglandins. However, few of them have been evaluated in clinical trials (144). Meloxicam, one of the NSAIDs, has been proved to have no benefit in reducing the rate of UO relapse, shortening the clinical episodes, and decreasing the severity of LUTS in two investigations (146, 147). Moreover, the potential of meloxicam to reduce blood flow to the kidneys and induce acute renal injury in cats, especially those with decreased water intake and possibly dehydration, further undermine the recommendation of meloxicam in clinical use (17, 29).

##### Treatment for Urethral Obstruction

Idiopathic UO is a common emergency occurring with higher frequency in male cats with FIC (148), secondary to urethral spasm and oedema (149). In addition to the medicines listed above, supportive fluid therapy is needed to correct electrolyte disturbances and stabilize cardiovascular and metabolic derangements (35, 150). Also, to relieve the obstruction, decompressive cystocentesis, retrograde urethral flushing and indwelling urethral catheterization with connection to a sterile closed collection system for a period of 24–48 h may be conducted due to different situations (9, 151). Further hospitalized medical management is usually required, see more details in Cosford's review (152). If UO recurs rapidly and frequently after initial management, surgery like perineal urethrostomy is recommended, see details in Nye's review (153).

#### Drugs for Chronic FIC

Chronic FIC refers to refractory FIC with recurrent episodes.

As stated before, the etiopathogenesis of FIC may relate to stress and anxiety in cats. Alprazolam, a benzodiazepine and anxiolytic, could be administered to subside clinical signs in cats with FIC (147). Since the increased concentrations of serotonin have been proved to have association with decreased anxiety in people and animal models (154), amitriptyline, one of tricyclic antidepressants (TCAs) may be beneficial in the treatment of FIC because it can increase serotonin content by inhibiting the reuptake of serotonin and NE and enhancing the neurotransmission of monoamine transmitters (9, 141). In addition to anti-depressant effects, TCAs also have anti-cholinergic, anti-adrenergic, anti-inflammatory, and analgesic effects (37). Side effects of TCAs include sedation, somnolence, weight gain, as well as liver dysfunction and urinary retention (37, 155). Also, amitriptyline should be wean off over a period of 1–2 weeks in case the clinical signs of FIC rebound (155). However, although amitriptyline has been proved beneficial in the long-term treatment of chronic FIC (155, 156), it may have little or no benefit for short-term resolution of clinical signs in cats with acute FIC (139, 141, 156).

Feline facial pheromone (FFP) may activate vomeronasal sensory neurons and regulate social behaviors in cats (17, 157). Feliway is a synthetic analog of F3 fraction of FFP, and it is developed to manage anxiety-related diseases, like FIC (4). Exposure to Feliway has been identified to have an anxiolytic effect in hospitalized cats with FLUTD (158), and shows a trend of reducing severe episodes and recurrences of FIC (159). But surprisingly, in a pilot study, no significant difference has been detected between the Feliway-treated group and placebo-treated cats with FIC (160), and recent reviews have questioned its efficacy of soothing cats (161–163). However, although unsupported by some studies, Feliway is still applied to cats with FIC in clinical practice, especially in refractory recurring cases (147).

A defective layer of endogenous GAGs covering the urinary bladder epithelium has been proposed as possible pathogenesis for FIC (17, 144, 164, 165). Clinical studies in human patients with IC/BPS has identified that oral or parenteral administration of GAG supplement could significantly alleviate symptoms (166–168). Therefore, using exogenous forms of GAG, including pentosan polysulfate sodium (PPS), glucosamine, chondroitin sulfate, heparan, and hyaluronic acid has been suggested in the treatment of cats with FIC (17, 37, 144, 160), because they may replace and repair damaged parts of the bladder wall as found in human medical research (169), and may also exhibit analgesic and anti-inflammatory effects (37). Side effects of both oral and intravesical GAGs are uncommon (160, 164, 170), but may include prolonged bleeding times and decreased appetite (37). PPS is a semi-synthetic sulfated proteoglycan, whose function and structure is similar to GAG (37, 169, 170). Cats treated with orally administered (171) and intravesically infused PPS (170, 172) make no more significant improvements than placebo group in both short-term and long-term follow-up. Based on current findings, PPS may not be an ideal medicine to reduce the recurrence rate and clinical signs in cats with FIC. N-acetyl glucosamine (NAG) is the precursor of GAG (37), and maybe more suitable to bind with urinary epithelium than other forms of GAGs (173). One randomized controlled study has confirmed that orally administrated NAG significantly increased plasma GAG concentrations in cats with FIC (173), which may help repair the damaged urothelium, and thus reduce the severity or relapse rate of LUTS in cats with FIC. However, in a well-designed study, although owner assessments indicated that NAG-treated cats with FIC had a slightly greater improvement compared to the placebo group, all groups achieved clinical benefit, and no significant difference was observed, suggesting a strong placebo effect (160). Also, in another pilot study, cats with FIC treated with intravesical instillation of a commercially available GAG solution, which contains NAG, chondroitin sulfate and hyaluronic acid, showed no differences compared with placebo treated group in terms of pain score and recurrence number in a 7-day period (164). Therefore, solid evidence is lacking for the clinical use of NAG for FIC. In addition, intravesical infusion of alkalinized lidocaine has been found to ameliorate clinical signs of IC/BPS in human patients (174), which suggests that it may be an alternative procedure for cats with FIC as well. But in a clinical trial, intravesical administration of lidocaine together with sodium bicarbonate had no apparent beneficial effect on decreasing recurrence of obstruction and severity of LUTS in cats with FIC (175). Nevertheless, although GAG supplementation is not recommended by some research studies, it is still used in some intractable cases, which do not respond well to other treatments. For pharmacological management of FIC, see Table 1.

**Table 1:** Drugs used in the management of FIC [adapted from Chew et al. (29)].

**Drug**	**Class**	**Mechanism of action**	**Indications**	**Suggested dosage**	**Potential adverse effects**
**Acute therapy**					
Acepromazine	Phenothiazine derivative	Inhibits alpha adrenergic receptors and dopamine brain receptors	Antispasmodic	0.05 mg/kg TID SC	Sedation, hypotension
Prazosin	Alpha adrenoceptor antagonist	Inhibits α1 adrenergic receptors	Antispasmodic	0.5 mg per cat BID PO	Sedation, hypotension
Phenoxybenzamine	Alpha adrenoceptor antagonist	Inhibits α1 and α2 adrenergic receptors	Antispasmodic	2.5 mg per cat BID PO	Sedation, hypotension
Dantrolene	Skeletal muscle relaxants	Inhibits the release of Ca^2+^ from the sarcoplasmic reticulum	Antispasmodic	0.5–2.0 mg/kg BID PO	Liver toxicity
Butorphanol	Synthetic partial opioid agonist	Activates κ1 receptors	Analgesia, acute episode	0.2–0.4 mg/kg TID PO or SC	Sedation
Buprenorphine	Synthetic partial opioid agonist	Activates μ receptors	Analgesia, acute episode	0.01–0.02 mg/kg BID to q8h PO or SC	Sedation
Fentanyl	Opioid agonist	Activates opioid receptors	Analgesia, acute episode	25 μg/h IV or fentanyl patches	Respiratory depression, bradycardia
Propantheline	Anticholinergics	Inhibits muscarinic receptors	Acute Episode, urge incontinence	0.25–0.5 mg/kg SID or BID PO	Liver toxicity
**Drug**	**Class**	**Mechanism of action**	**Indications**	**Dosage**	**Potential adverse effects**
**Chronic therapy**					
Amitriptyline	Tricyclic antidepressant	NE reuptake inhibition; central and peripheral anticholinergic activity; antagonism of the H1 receptor; 5-HT reuptake inhibition; glutamate and Na^+^ channel receptor antagonist	Chronic FIC	0.5–2.0 mg/kg per cat SID PO	Sedation, anti-cholinergic effects, weight gain, urine retention, urolith formation
Clomipramine	Tricyclic antidepressant	NE and serotonin reuptake inhibition	Chronic FIC, urine spraying	0.5 mg/kg SID PO	Sedation, anti-cholinergic effects
Fluoxetine	Selective serotonin reuptake inhibitor	Strong inhibitor of serotonin reuptake and very weak inhibitor of NE reuptake	Chronic FIC, urine spraying	1 mg/kg SID PO	Rare: Decreased food intake, vomiting, lethargy
Buspirone	Selective serotonin reuptake inhibitor	Blocks pre- and post-5-HT1A receptors; downregulates 5-HT2 receptors; moderate affinity for D2-dopamine brain receptors	Chronic FIC, urine spraying	2.5–5.0 mg per cat BID PO	Rare: Sedation, other neurologic effects
Alprazolam	Benzodiazepine—anxiolytic	Facilitates GABA in the CNS—binding to GABA_A_ receptors	Chronic FIC, urine spraying	0.0125–0.025 mg/kg TID PO; 0.125–0.25 mg/cat BID PO (low dose first to evaluate for paradoxical excitement)	Sedation, increased appetite, ataxia; paradoxical excitement (rare)
F3 fraction of feline facial pheromone	Synthetic pheromone	Alters emotional state of the animal *via* the limbic system and hypothalamus	Anxiety-related behaviors, chronic FIC	One spray in affected area once daily as needed, or room diffuser	None reported
Pentosan polysulfate sodium	Glycosaminoglycan supplement	replace and repair damaged parts of the bladder wall	Chronic FIC	8–16 mg/kg per cat BID PO	Rare: Decreased food intake, prolonged bleeding times, insulin resistance
N-acetyl glucosamine	Glycosaminoglycan supplement	replace and repair damaged parts of the bladder wall	Chronic FIC	125–250 mg per cat SID PO	Rare: Vomiting, diarrhea

#### Other Possible Therapeutic Compounds for FIC

Some ω-3 fatty acids like eicosapentaenoic acid (EPA) and docosahexaenoic acid (DHA), and β-carotene, and vitamin E, especially the major bioactive form α-tocopherol, are purportedly anti-oxidant and anti-inflammatory agents (176, 177). Therapy protocol adding these supplements is hypothesized to decrease free radical-induced bladder inflammation caused by oxidative stress, and thus reduce clinical signs and recurrent episodes in cats with FIC (176). A commercially available prevention cat food enriched with EPA, DHA and other antioxidants has been found to significantly reduce recurrent episodes with a combination of LUTS, as well as with individual LUTS of hematuria, dysuria, and stranguria, but not pollakiuria and periuria in cats with FIC in a long-term follow-up study (140). L-tryptophan, a precursor for serotonin synthesis, is thought to decrease signs of anxiety by increasing serotonin activity, and has been detected to generate positive influence on cats with stress-related disorders (178). Also, α-casozepine, a hydrolyzed casein derived from milk, has been associated with significant alleviation of stress in anxious cats (178, 179). The mechanism of α-casozepine to exert its anxiolytic effects may be mediated through the benzodiazepine site of the γ-aminobutyric acid receptor (179, 180). Additionally, a therapeutic urinary stress diet containing antioxidants has also been identified to reduce the recurrence rate of FIC in the short term (181). Therefore, nutritional intervention added with these therapeutic supplements is promising for the treatment of FIC (17, 39).

Furthermore, some natural and safe herbal compounds are advised for the management of FIC. Cranberry extract can produce an anti-inflammatory effect by inhibiting the activity of cyclooxygenase-2 and suppressing several pro-inflammatory interleukins (182, 183). And the high proanthocyanidins content of cranberry may act to prevent toxic factors from attaching to the urothelium. In a recent preliminary study, cranberry extract has been identified to be effective in reducing LUTS in cats with FIC (184). Additionally, the effect of traditional Chinese medicines San Ren Tang, Wei Ling Tang and Alisma to manage FLUTD in cats have been investigated in a randomized, placebo-controlled, crossover-designed study, but no significant differences have been observed between treatment and control group (185). However, future scientifically-designed studies with longer treatment duration, sufficient dosage, as well as in a larger sample size are warranted to further decide the efficacy of traditional Chinese medicines.

### Behavioral Therapy

A large number of studies have revealed that poor adaptive ability to environmental and physiological stressors may play an important role in the pathogenesis of cats with FIC (34, 64, 85). Accordingly, tailored behavior therapy is essential to reduce and remove conflicts, and decrease the activation risk of the SRS (28), possibly by changing the cat's perception of stressors through the EMGEX mechanism (99, 186). Multimodal environmental modification (MEMO), or feline environmental enrichment (FEE), defined as the addition of one or more factors to a relatively impoverished environment to improve the physical and psychological welfare of cats (187), has been proved to act successfully in improving LUTS, as well as other clinical signs of comorbid disorders in cats with FIC (28). Also, when hospitalized, cats may feel more safe and cope better with the confinement if the environment is enriched (39). Therefore, MEMO is suggested as the primary therapy for cats with FIC, usually before pharmacological intervention (9, 28, 187). MEMO could include physical, occupational, social, sensory, and nutritional approaches (187, 188), and applying MEMO before or at the same time with medicines or other management methods of FIC is likely to enhance the overall efficacy, so a comprehensive treatment programme is needed to reach the best therapeutic effect (92, 99). However, a recent study has discovered that the majority of cats' living environments are barely moderately enriched, owning to the fact that most guardians lack awareness of MEMO, or they just lack the patience and persistence to implement enrichment measures (187). So, in veterinary practice, clinicians should educate and help clients formulate a SMARTR (specific, measurable, action-oriented, realistic, timely and rewarded) environmental enrichment plan based on the behavioral history, current surrounding and individual preference of the diseased cat (28, 188).

There are five pillars that constitute a healthy feline environment (156, 189, 190), and these are core requirements for pet owners and veterinarians to examine and exert in the management of cats with FIC.

First and foremost, since cats are solitary hunters who tend to avoid and evade potential dangers, a safe place should be provided for every cat at home, during transport, and in the veterinary hospital (39, 156, 189). A recent study has certified that providing hiding boxes for cats assists them in managing stress and adapting to the shelter environment faster (191). A safe place could be cardboard boxes, carriers, and most likely, elevated vantage locations such as hammocks, perches and shelves, which allow cats to monitor their surroundings, withdraw from conditions they perceive threatening or unfamiliar, and attain a sense of control, as well as a feeling of isolation and protection (39, 189). In addition, if the cat with FIC is hospitalized, covering a blanket in front of the cage may help reduce its anxiety (39), while if the cat lives in a multi-cat household, make sure every safe place has multiple entries so that access cannot be easily blocked by another cat (189).

In the second place, although cats can either live alone or in social groups, they do not like to be challenged by other cats in regards to territory and resources. If space and resources are insufficient, offensive or defensive conflicts will exist between cats, and the threatened cats are more likely to develop FIC (39). Therefore, multiple and separated key environmental resources, including food, water, toileting, resting and playing areas, should be provided (39, 189, 192). A widely-accepted standard rule is to provide as many resources as there are cats plus an additional one (17, 192). Additionally, these key resources should be available in various locations, so as to allow every individual cat to express preference, and prevent cats live in a multi-cat household from encountering and competing with each other (156, 189). In hospital cages, resources should also be spread out, for example, the resting and hiding areas should be separated from the feeding area (189). Moreover, the management of litter boxes in cats with FIC should consider the boxes' cleanness, size and style, as well as litter type (193, 194). Cats prefer cleaned toileting area, so the litter should be scooped at least once daily and replaced completely once per week (194). Many researchers suggest a larger size of litter boxes is better for cats (17, 156, 194). However, choices for the covered or uncovered type of litter boxes differ from different cats (17, 156, 194). Studies have identified that cats show no special preference in the scent of litter (4). In general, clumping clay litter with a sand-like texture is appealing to most cats (17, 194). Dietary management of FIC is also of great importance, and its main goal is to increase water intake and promote urine dilution of the affected cat, so that toxic molecules will be excreted in time (192, 195, 196), thus prevent further damage of the bladder and body. Keeping water fresh and cleaning food and water bowls regularly are suggested (39). Additionally, adding flavoring to water, adding water or broth to dry food, canned food and high sodium diets are recommended for cats with FIC to increase their water intake (144, 196), dilute their urine (160), and could result in a great decrease in recurrence rate (195). Also, since cats may prefer a water source that can be investigated, water fountains and dripping faucets with movements may be beneficial (39, 144, 196). However, one study has found that water fountains may not act as successfully as we thought, which means individual preference of different cats should be taken into consideration (196), and a tailored maneuver to encourage their water intake should be determined for every diseased cat.

Thirdly, play and predatory behaviors of cats should be encouraged and allowed to perform regularly (189). Cats retain the instinct to locate, capture and kill prey, but the indoor lifestyle of most cats in modern society limits their activities and suppresses their innate behaviors, which may result in stress-related disorders, including FIC (189). Therefore, cats must engage in play and predatory behaviors. This could be achieved by hiding food in diverse corners of the house or in food puzzles for cats to explore and exercise, providing a variety of toys, like laser points, feathers and ropes, for cats to chase and capture, and increasing play-based interaction with the cat owner and with other socially compatible cats (9, 39, 189). Also, the stimulation methods should be based on cats' personal preference and toys should be rotated and replaced regularly to sustain cats' interest (39).

Furthermore, cats are companion animals and a positive, consistent, and predictable interaction need to be built between cats and their caregivers (189). Social preferences vary from cats to cats, and if disregarded, stress-related diseases like FIC will occur. The social preferences of cats can be influenced by their genetics, early rearing experiences and life experiences (93). The most critical age for kittens to socialize with and adapt to human beings is during 2–7 weeks, and the cats who have positive handling experiences during this formative period will cope with stress better and display less fear in future (189), so an early friendly rearing is recommended. Additionally, since a majority of cats prefer a high frequency and low intensity level of social contact with their guardians, a daily 10–15 min of interaction is advised to strengthen human-cat bonds and promote the physical and mental welfare of cats (187, 189).

Lastly, an environment that respects cats' senses, especially the sense of smell, should be provided (187, 189). Cats mainly depend upon olfactory and chemical information. They evaluate their surroundings, establish the boundaries of their core living areas, and maximize their senses of security and comfort by scratching and rubbing their faces or bodies on objects (189). Therefore, guardians should let cats express and receive chemical signals at their will, and try not to interfere with cats' olfactory and pheromonal information. In addition, smells, noises, items and animals that cats consider as unfamiliar and strange should be limited to minimum in case threatening conditions stress cats (189). In addition, certain odors such as catnip (Nepeta cataria) or lavender, along with video and audio simulations, have been proposed as entertainment and enrichment for the life of domestic cats (187).

In short, effective MEMO for cats with FIC should at least include minimization of conflicts, provision of all necessary resources, refinement of interactions with owners, and gradual changes in every aspect (17, 39).

## Prognosis

Signs in LUT are easy to recur in the majority of FLUTD cats irrespective of the identified etiology (197), and the affected cats may present with different causes in different recurrent episodes, for example, cats with FIC are predisposed to develop urolithiasis or bacterial cystitis in their later relapses (197, 198). Nevertheless, no significant difference in the number of recurrent episodes has been observed between cats diagnosed as different classifications of FLUTD (197). The recurrence rate of FIC varies dramatically between diverse studies. One study proposed that a lower incidence of recurrence may be associated with the increased age of cats with FIC (141). A previous study which included only idiopathic UO and UO relapses, has reported a 36% (8/22 cats) recurrence rate after monitoring for 3–728 days (median 17 days) (199). Additionally, in three other studies, the recurrence rate of obstructive FIC has been found to be 17.1% (170), 18.8% (164), and 24.3% (146) over a 4-, 7-, and 180-day period, respectively. However, when cats with FIC with and without UO and recurrent episodes with and without obstructive LUTS were included, the recurrence rate of FIC has been proposed to be 65% (160), 61.5% (197), and 52% (200) within a follow-up length of 6 months, 0.5–138 months, and 10–16 years, respectively. Compared to the former ones, the latter data regarding to the relapse risk of FIC are higher, and the broad definition, along with the longer observation term may be responsible for the difference.

Similarly, there is a great variation in the mortality rate of FIC in different studies. Some cats with FIC may end up being euthanized because their owners could not endure the frequent relapses and the chronic course of the disease (1). Also, due to the interrelation mechanisms that exist in FLUTD cats, some cats with FIC may die because of FLUTD-related factors, such as urolithiasis, urethral plugs and UTI (4). The mortality related to FLUTD among cats with different etiologies was reported to be 5% (197), while the mortality rate of FIC exclusively was reported to be 12.5% (29) and 20% (200) in two retrospective studies. The mortality rate of obstructive FIC has been suggested to be 26% (199). Therefore, more than 70% of the cats with FIC could survive and even live longer than 10 years (200).

To sum up, despite the high recurrence rate, the mortality rate of FIC is relatively low. Although hard to recover completely, the number of recurrent episodes and the severity of clinical symptoms could be decreased when applying appropriate medicines and environment enrichment procedures, so the long-term prognosis for cats diagnosed with FIC is quite positive.

## Conclusion

In veterinary practice all over the world, FIC is a common acute or chronic disease with frequent recurrences, and it is most likely to occur in relatively young and overweight cats with a low level of activity. Various factors will increase the risk of developing FIC, and most of them are stress-related. The exact etiopathogenesis of FIC has not been defined yet, but many hypotheses have been proposed, and the one relating to neuroendocrine factors appears to be most promising. In addition to LUTS like dysuria, stranguria, hematuria, pollakiuria, and periuria, comorbid disorders of other body systems also occur in cats with FIC. Therefore, the tentative term “pandora syndrome” is used to represent this complex systemic disease. The diagnosis of FIC is still a process of excluding other forms of FLUTD. Although some potential diagnostic biomarkers have been identified, they are not sensitive and specific enough for clinical application. The management of FIC is usually long-term management that needs the compliance and dedication of pet owners. The major treatment for FIC should be MEMO based on the behavioral history and personal preferences of the affected cat. Psychopharmacological management, GAG supplements, and some therapeutic additives are recommended in refractory cases that do not respond to other medicines well.

However, there are still many aspects of the disease that need to be discussed in future studies. Firstly, the specific pathophysiology of FIC needs to be clarified. Also, risk factors of FIC need to be investigated and evaluated in a larger cohort including diseased cats with diverse lifestyles from different parts of the world. Additionally, the sensitivity and specificity of potential diagnostic biomarkers need to be tested, and hopefully, some of them could be made available in clinical use, making the diagnosis of FIC easier. Moreover, the efficacy and safety of some medicines recommended for the treatment of cats with FIC need to be further examined in randomized, double-blinded, placebo-controlled, prospective clinical trials with a sufficiently large sample size.

## Author Contributions

All authors listed have made a substantial, direct, and intellectual contribution to the work and approved it for publication.

## Funding

This work was supported by grant from the Beijing Zhongnongda Veterinary Hospital Co., Ltd.

## Conflict of Interest

The authors declare that the research was conducted in the absence of any commercial or financial relationships that could be construed as a potential conflict of interest.

## Publisher's Note

All claims expressed in this article are solely those of the authors and do not necessarily represent those of their affiliated organizations, or those of the publisher, the editors and the reviewers. Any product that may be evaluated in this article, or claim that may be made by its manufacturer, is not guaranteed or endorsed by the publisher.

## Glossary

acetylcholine (Ach)

adenosine triphosphate (ATP)

adrenocorticotropic hormone (ACTH)

anti-proliferative factor (APF)

bacterial urinary tract infection (UTI)

bovine herpesvirus type 4 (BHV-4)

brain-derived nerve growth factor (BDNF)

central nervous system (CNS)

central threat response system (CTRS)

corticotropin releasing factor (CRF)

C-X-C motif chemokine ligand 12 (CXCL12)

complement 4a (C4a)

docosahexaenoic acid (DHA)

eicosapentaenoic acid (EPA)

endothelial growth factor (EGF)

epigenetic modulation of gene expression (EMGEX)

feline calicivirus (FCV)

feline environmental enrichment (FEE)

feline facial pheromone (FFP)

feline foamy virus (FFV)

feline idiopathic cystitis (FIC)

feline lower urinary tract disease (FLUTD)

fms-related tyrosine kinase 3 ligand (Flt3L)

feline urologic syndrome (FUS)

glycosaminoglycan (GAG)

hemoglobin-binding endothelial growth factor (HB-EGF)

hypothalamic-pituitary-adrenal (HPA)

interstitial cystitis /bladder pain syndrome (IC/BPS)

interleukin 12 (IL-12)

interleukin 18 (IL-18)

locus coeruleus (LC)

lower urinary tract (LUT)

lower urinary tract disease (LUTD)

lower urinary tract signs (LUTS)

multimodal environmental modification (MEMO)

N-acetyl glucosamine (NAG)

nerve growth factor (NGF)

nitric oxide (NO)

non-steroidal anti-inflammatory drug (NSAID)

norepinephrine (NE)

nuclear factor-κB p65 (NF-κB p65)

pentosan polysulfate sodium (PPS)

p38 mitogen-activated protein kinase (p38 MAPK)

substance P (SP)

sympathetic nervous system (SNS)

Tamm-Horsfall protein (THP)

trefoil factor 2 (TFF2)

tricyclic antidepressant (TCA)

tyrosine hydroxylase (TH)

urethral obstruction (UO)

zonula occludens-1 (ZO-1)α2 adrenergic receptor (α2-AR)
